# Low bone mineral density and its related factors in adults with congenital heart disease in Vietnam: A cross‐sectional study

**DOI:** 10.1002/hsr2.732

**Published:** 2022-08-07

**Authors:** Thanh‐Huong Truong, Mai‐Ngoc Thi Nguyen, Ngoc‐Thanh Kim, Thuy‐Hoa Thi Nguyen, Doan‐Loi Do, Thanh‐Tung Le, Hong‐An Le

**Affiliations:** ^1^ Department of Cardiology Hanoi Medical University Hanoi Vietnam; ^2^ Vietnam National Heart Institute Bach Mai Hospital Hanoi Vietnam

**Keywords:** bone mineral density, cardiovascular disease, congenital heart disease, polycyemia

## Abstract

**Background and Aims:**

Recent studies have highlighted the increased risk of low bone mineral density (BMD) in adults with cardiovascular disease. However, little is known about BMD in adults with congenital heart disease (CHD), particularly in developing countries. We hypothesized that factors related to BMD would lead to a high prevalence of low BMD in adults with CHD. This study aimed to determine the prevalence of low BMD and its related factors in Vietnamese adults with CHD.

**Methods:**

We conducted a cross‐sectional study of 73 adults diagnosed with CHD in Vietnam. Low BMD was classified based on their site‐specific *Z*‐scores and *T*‐scores at the posteroanterior lumbar spine and left proximal femur. Logistic regression analyses were performed to evaluate factors related to low BMD.

**Results:**

Low BMD was confirmed in one‐third of the adults with CHD. There were trends of more bone loss in certain parts of the body than in others, with the prevalence of low BMD at the sites of the lumbar vertebrae (L1‒L4) and left proximal femur (femoral neck, trochanteric femur, and intertrochanteric area) of 43.9%, 31.8%, 28.8%, 33.3%, 8.8%, 1.5%, and 6.1%, respectively. The prevalence of low BMD in the lumbar spine was significantly higher than that in the left proximal femur (34.3% vs. 2.9%, *p* < 0.001). Moreover, the prevalence of low BMD was significantly higher in adults with CHD than in those without polycythemia and vitamin D deficiency (55.6% vs. 20.9%, *p* = 0.001 and 46.2% vs. 19.4%, *p* = 0.002, respectively). A stratified multivariate logistic regression analysis revealed that low BMD was associated with polycythemia (odds ratio: 4.72; 95% confidence interval: 1.64–13.58, *p* = 0.004).

**Conclusions:**

Low BMD is common among adults with CHD in Vietnam and related to polycythemia.

## 1 INTRODUCTION

Congenital heart disease (CHD), the presence of structural heart and great vessel defects at birth, has a prevalence of approximately 1 per 100 live births worldwide. As longevity and healthcare quality improve, many patients with CHD now survive until adulthood, markedly changing the disease pattern and requiring management in adulthood.^1^ According to updated guidelines, such management should be comprehensive.^2^ In particular, due to circulation problems, individuals with CHD also develop from bone health issues and even fractures.^3^

Osteoporosis involves a gradual decrease in bone mineral density (BMD) and microarchitectural deterioration of bone tissue, leading to bone fragility. The gold standard for osteoporosis diagnosis is BMD, which is measured by dual‐energy X‐ray absorptiometry scans (DXA). Osteoporosis has a complex pathophysiology that is determined by factors such as sex, age, hormones, nutrition, and lifestyle.^4^ Poor bone quality imposes a significant public health burden because it increases the risk of mortality and morbidity.^5^

It is hypothesized that CHD shares many biological risk factors with low bone quality. Recent evidence suggests that low bone quality, including osteoporosis, is common among patients with CHD.^6^ Currently, studies on bone quality in adults with CHD, particularly in Asian and developing countries, are lacking. Thus, this study aimed to determine the prevalence of low BMD and its related factors in adults with CHD in Vietnam.

## 2 MATERIALS AND METHODS

### 2.1 Study design

From May 2019 to September 2020, we conducted a cross‐sectional study of adults with CHD who were followed up at the Vietnam National Heart Institute, a national referral cardiovascular center specializing in CHD.

### 2.2 Patient selection

We selected patients who were confirmed to have CHD on cardiac imaging. All patients underwent transthoracic echocardiography performed by a cardiologist with cardiac sonography certification at least once. If diagnosis was unclear, patients underwent another form of imaging, such as transesophageal echocardiography, chest computed tomography, cardiac magnetic resonance imaging, and right heart catheterization. The final diagnosis was confirmed after consultation with a CHD specialist. Patients were included if their BMD was determined by DXA, and if they were aged ≥18 years.

We excluded patients with any of the following characteristics: peri‐ and postmenopause; acute medical status for the past 3 months, and known disorder(s) affecting bone metabolism (e.g., thyroid disorder, adrenal hormone disorders, systemic disease, cirrhosis, or severe renal failure with estimated glomerular filtration rate [eGFR] <30 ml/min/1.73 m^2^); and those with a history or current use of drugs associated with osteoporosis or long‐term bone effects, such as corticosteroids, aromatase inhibitors, gonadotropin‐releasing hormone agonists, androgen‐deprivation therapy, injection contraception, medroxyprogesterone acetate, selective serotonin reuptake inhibitors, antiepileptic drugs, calcineurin inhibitors, and chemotherapy agents. We also excluded those who were pregnant or breastfeeding.

### 2.3 Sample size

We calculated the sample size for this cross‐sectional study using the following formula: $N=\frac{Z_{1-\alpha /2}^{2}\;P(1-P)}{d^{2}}$, where *N* is the sample size, *Z* is the statistic corresponding to the confidence level, *P* is the expected prevalence, and *d* is the precision. We selected *P* from a recent study that found an osteoporosis prevalence of 43% among Vietnamese women aged ≥50 years.^7^ With a 95% confidence interval (CI) and precision of 0.15, adding 15% for missing data, the appropriate sample size was calculated as 73.

### 2.4 Data collection

Demographic and clinical information were obtained at consultation. We used the International Classification of Disease, 10th revision, for CHD diagnosis. Young adults were defined to be aged between 18 and 35 years.^8^ The New York Heart Association (NYHA) functional classification was used to stratify heart failure severity. Saturation of peripheral oxygen (SpO_2_) was measured using a Life Scope BSM3500 (Nihon Kohden). Cyanosis was defined as a SpO_2_ ≤92% in room air. Pulmonary artery hypertension (PAH) was defined as a systolic pulmonary artery pressure ≥50 mmHg as estimated from tricuspid valve regurgitation on echocardiography. Anemia was defined as a hemoglobin level <12.0 g/dL in women and <13.0 g/dL in men. Polycythemia was defined as a hemoglobin level ≥185 g/L in men or ≥165 g/L in women with a hematocrit value ≥51% in men and ≥48% in women.

Biochemical testing was performed at the Department of Biochemistry, Bach Mai Hospital, according to the ISO 15189:2007 standards. Renal dysfunction was defined as an eGFR ≤90 ml/min/1.73 m^2^. Liver dysfunction was diagnosed if plasma total bilirubin and aspartate transaminase and/or alanine transaminase levels were elevated more than the reference ranges for laboratory values (17.1 µmol/L, 37 U/L, and 41 U/L, respectively). Plasma total calcium levels were measured by Cobas 8100 (Roche), while the levels of plasma 25‐hydroxyvitamin D (25[OH]D) and parathyroid hormone (PTH) were measured using Architect i2000SR (Abbott). We defined cutoff points of vitamin D deficiency as plasma 25(OH)D levels <20 ng/mL, severe vitamin D deficiency as plasma 25(OH)D levels <12 ng/mL, hypocalcemia as plasma total calcium levels <2.15 mmol/L, and hyperparathyroidism as plasma PTH levels >7.2 pmol/L.

BMD was evaluated at the posteroanterior lumbar spine (lumbar vertebrae, L1‒L4) and left proximal femur (femoral neck [neck], trochanteric femur [troch], and intertrochanteric area [inter]) using the Discovery DXA system software version 13.5.4 (Hologic) for baseline scans. Site‐specific *Z*‐scores were calculated as the number of standard deviations (SDs) away from the average value of the reference group, with the following formula: *Z*‐score = (BMD‐expected BMD)/SD. Site‐specific *T*‐scores were calculated for participants aged >50 years. The *T*‐score was the number of SDs of the patient's measured BMD from a reference peak BMD (mean BMD of sex‐matched young adults) calculated using the following formula: *T*‐score = (patient's BMD‐peak BMD)/SD. In premenopausal women and men aged <50 years, low BMD was defined as a site‐specific *Z*‐score ≤−2.0, as described by the recommendations of the International Society for Clinical Densitometry. In men aged >50 years, low BMD was defined as a site‐specific *T*‐score ≤−2.5 (osteoporosis) according to the World Health Organization criteria. Notably, a site‐specific *T*‐score between −1 and −2.5 was considered to indicate osteopenia.^9^

### 2.5 Outcome measures

The primary outcome measures were bone characteristics (BMD, *T*‐score, and *Z*‐score) and low BMD prevalence at the evaluated site in adults with CHD overall and in subgroups. The secondary outcomes were low BMD‐related factors in these patients.

### 2.6 Statistical analysis

Data were analyzed using SPSS version 22.0 (IBM). Continuous variables are described as mean (SD, 95% CI) for normally distributed data or median (interquartile range) for non‐normally distributed data. Numbers and percentages are used to describe nominal variables. Among subgroups, normally distributed continuous variables were compared using Student's *t*‐test or Mann–Whitney *U *test, while nominal variables were compared using *χ* ^2^ test or Fisher's exact test. Linear regression was used to model the relationship between age and BMD at the lumbar spine and left proximal femur for adults with CHD. Univariable and multivariable forward logistic regression models were used to evaluate associations between clinical and paraclinical characteristics and low BMD in adults with CHD through the forward stepwise method (likelihood ratio). In all analyses, statistical significance was defined as a two‐tailed *p* < 0.05.

## 3 RESULTS

### 3.1 Patient characteristics

The characteristics of 73 adult patients with CHD are summarized in Table 1. There were 42 premenopausal women, 29 men aged ≤50 years, and 2 men aged >50 years. A high proportion of adults with CHD were aged over 35 years and underweight and had cyanosis, polycythemia, and NYHA Class III‒IV. Vitamin D deficiency, hyperparathyroidism, and hypocalcemia were common and similarly prevalent between the sexes. Notably, our study did not contain any adults with CHD having a history of smoking, alcoholism, diabetes, anticoagulant use, and/or calcium supplement use. Supporting Information: Table 1 shows that isolated atrial septal defect and ventricular septal defect were the most common cardiac congenital malformations in patients. Approximately, 50% of adults with CHD had PAH, and almost one‐quarter had complex CHD.

**Table 1:** Clinical characteristics in adults with congenital heart disease

Clinical characteristics		Total (*N *= 73)	Women (*N* = 42)	Men (*N* = 31)	*p*
Age (years)	Mean (SD, 95% CI)	32.4 (10.1, 30.1–34.8)	31.7 (8.8, 29.0–34.5)	33.4 (11.8, 29.0–37.7)	0.81*
	Age >35 years (%, *n*)	32.9 (24)	31.0 (13)	35.5 (11)	0.68
BMI (kg/m^2^)	Mean (SD, 95% CI)	19.0 (2.4, 18.4–19.5)	18.9 (2.3, 18.2–19.6)	19 (2.5, 18.1–19.9)	0.85
	Underweight	42.5 (31)	35.7 (15)	51.6 (16)	0.17
SpO_2_ (%)	Mean (SD, 95% CI)	89.5 (9.7, 87.2–91.8)	92.8 (8.2, 90.2–95.3)	85.1 (10, 81.4–88.7)	<0.001*
	Cyanosis (%, *n*)	46.6 (34)	28.6 (12)	71.0 (22)	0.001
Plasma 25[OH]D level (ng/ml)	Mean (SD, 95% CI)	19.6 (6.2, 18.1–21.0)	19.7 (5.2, 18.1–21.3)	19.4 (7.4, 16.7–22.1)	0.85
	Vitamin D deficiency (%, n)	53.4 (39)	52.4 (22)	54.8 (17)	0.84
	Severe vitamin D deficiency (%, *n*)	4.1 (3)	2.4 (1)	6.5 (2)	0.57^**^
Plasma PTH level (pmol/L)	Mean (SD, 95% CI)	8.4 (7.0, 6.8–10.0)	7.0 (4.2, 5.7–8.4)	10.2 (9.4, 6.8–13.7)	0.08*
	Hyperparathyroidism (%, *n*)	41.1 (30)	33.3 (14)	51.6 (16)	0.12
Plasma total calcium level (mmol/L)	Mean (SD, 95% CI)	2.25 (0.11, 2.23–2.28)	2.27 (0.09, 2.24–2.3)	2.23 (0.14, 2.18–2.28)	0.16
	Hypocalcemia (%, *n*)	30.1 (22)	22.4 (9)	41.9 (13)	0.06
Anemia (%, *n*)		12.3 (9)	19.0 (8)	3.2 (1)	0.07^**^
Polycythemia (%, *n*)		39.7 (29)	26.2 (11)	58.1 (18)	0.006
NYHA Class III–IV (%, *n*)		32.9 (24)	21.4 (9)	48.4 (15)	0.02
PAH (%, *n*)		46.6 (34)	42.9 (18)	51.6 (16)	0.46
Renal dysfunction (%, *n*)		34.2 (25)	28.6 (12)	41.9 (13)	0.23
Liver dysfunction (%, *n*)		5.5 (4)	0	12.9 (4)	0.03^**^

*Note*: Using Student's t‐test or *Mann–Whitney U test.

Using *χ* ^2^ test or **Fisher's exact test.

Abbreviations: 25[OH]D, 25‐hydroxyvitamin D; BMI, body mass index; CI, confidence interval; NYHA, New York Heart Association; PAH, pulmonary artery hypertension; PTH, parathyroid hormone; SD, standard deviation; SpO_2_, saturation of peripheral oxygen.

### 3.2 BMD in adults with CHD

**Figure 1:**
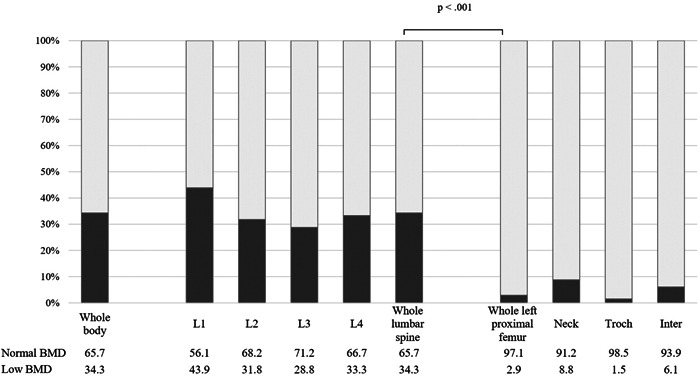
Prevalence of low bone mineral density (BMD) in adults with congenital heart disease, using *χ* ^2^ test. L, lumbar vertebrae.

The prevalence of low BMD is shown in Figure 1. Additionally, the overall prevalence of low BMD in the lumbar spine was significantly higher than that in the left proximal femur (34.3% vs. 2.9%, *p* < 0.001, respectively).

Table 2 summarizes the distribution of BMD in adults with CHD. Additionally, in both sexes, the *T*‐ and *Z*‐scores were low at any site of the lumbar spine (<−1.0) but in the normal range (>−1.0) at the left proximal femur. The overall mean BMD, *T*‐score, and *Z*‐score of the lumbar spine and left proximal femur were similar, except for the mean BMD at the trochanteric femur in women, which was significantly lower than that in men.

**Table 2:** Dual‐energy X‐ray absorptiometry variables in adults with congenital heart disease

Variables		Total				Women				Men			*p*
		Mean (SD)	95% CI	*n*		Mean (SD)	95% CI	*n*		Mean (SD)	95% CI	*n*	
*Lumbar spine*													
BMD, g/cm^2^	L1	0.784 (0.113)	0.758; 0.811	73		0.779 (0.118)	0.742; 0.815	42		0.792 (0.107)	0.753; 0.831	31	0.62
	L2	0.847 (0.117)	0.82; 0.875	73		0.843 (0.124)	0.804; 0.882	42		0.853 (0.109)	0.813; 0.893	31	0.72
	L3	0.891 (0.118)	0.864; 0.919	73		0.888 (0.126)	0.848; 0.927	42		0.896 (0.108)	0.857; 0.936	31	0.76
	L4	0.894 (0.135)	0.862; 0.925	73		0.892 (0.142)	0.848; 0.936	42		0.896 (0.128)	0.829; 0.918	31	0.91
	Full	0.859 (0.116)	0.832; 0.886	73		0.853 (0.125)	0.814; 0.892	42		0.867 (0.104)	0.829; 0.905	31	0.63
*T*‐score	L1	−1.83 (1.11)	−2.08; −1.57	73		−1.77 (1.0)	−2.09; −1.46	42		−1.9 (1.25)	−2.35; −1.44	31	0.64
	L2	−1.58 (1.09)	−1.83; −1.32	73		−1.49 (1.11)	−1.83; −1.14	42		−1.7 (1.07)	−2.09; −1.31	31	0.4
	L3	−1.59 (1.09)	−1.84; −1.33	73		−1.6 (1.17)	−1.97; −1.24	42		−1.57 (1.0)	−1.94; −1.2	31	0.9
	L4	−1.47 (1.19)	−1.75; −1.19	73		−1.41 (1.27)	−1.8; −1.02	42		−1.55 (1.11)	−1.96; −1.15	31	0.61
	Full	−1.64 (1.04)	−1.88; −1.4	73		−1.6 (1.11)	−1.94; −1.26	42		−1.69 (0.96)	−2.05; −1.34	31	0.71
*Z*‐score	L1	−1.74 (1.05)	−2.0; −1.48	66		−1.57 (1.02)	−1.9; −1.24	39		−1.99 (1.06)	−2.4; −1.57	27	0.11
	L2	−1.42 (1.07)	−1.68; −1.15	66		−1.24 (1.13)	−1.61; −0.88	39		−1.67 (0.95)	−2.04; −1.29	27	0.12
	L3	−1.41 (1.06)	−1.67; −1.15	66		−1.33 (1.2)	−1.71; −0.94	39		−1.53 (0.86)	−1.87; −1.18	27	0.46
	L4	−1.23 (1.16)	−1.51; −0.94	66		−1.14 (1.29)	−1.56; −0.72	39		−1.35 (0.95)	−1.49; −0.69	27	0.48
	Full	−1.51 (1.04)	−1.75; −1.26	70		−1.45 (1.18)	−1.82; −1.08	42		−1.59 (0.8)	−1.9; −1.28	28	0.59
*Left proximal femur*													
BMD, g/cm^2^	Neck	0.738 (0.217)	0.683; 0.776	73		0.752 (0.101)	0.721; 0.784	42		0.72 (0.313)	0.605; 0.835	31	0.88*
	Troch	0.694 (0.102)	0.67; 0.718	73		0.668 (0.076)	0.644; 0.691	42		0.73 (0.121)	0.686; 0.775	31	0.008
	Inter	0.976 (0.137)	0.944; 1.007	73		0.963 (0.118)	0.926; 1.0	42		0.992 (0.16)	0.912; 0.98	31	0.32*
	Full	0.893 (0.119)	0.865; 0.921	72		0.876 (0.1)	0.845; 0.907	42		0.917 (0.14)	0.865; 0.969	30	0.15
*T*‐score	Neck	−0.77 (1.04)	−1.01; −0.53	73		−0.72 (0.94)	−1.01; −0.43	42		−0.84 (1.18)	−1.27; −0.41	31	0.63
	Troch	−0.11 (0.92)	−0.32; 0.11	73		−0.1 (0.78)	−0.34; 0.15	42		−0.12 (1.09)	−0.52; 0.28	31	0.9
	Inter	−0.7 (0.87)	−0.91; −0.5	73		−0.65 (0.81)	−0.9; −0.4	42		−0.77 (0.97)	−1.12; −0.42	31	0.57
	Full	−0.36 (0.96)	−0.59; −0.14	72		−0.25 (0.89)	−0.53; 0.03	42		−0.52 (1.06)	−0.62; −0.11	30	0.24
*Z*‐score	Neck	−0.53 (1.08)	−0.79; −0.26	68		−0.47 (0.99)	−0.78; −0.15	40		−0.61 (1.22)	−1.09; −0.14	28	0.58
	Troch	0.02 (0.9)	−0.2; 0.24	66		0.05 (0.72)	−0.19; 0.28	39		−0.02 (1.13)	−0.1; 0.32	27	0.76
	Inter	−0.61 (0.86)	−0.83; −0.4	66		−0.51 (0.79)	−0.76; −0.25	39		−0.77 (0.95)	−1.15; −0.39	27	0.22
	Full	−0.3 (0.95)	−0.53; −0.07	69		−0.18 (0.9)	−0.46; 0.11	42		−0.5 (1.01)	−0.9; −0.1	27	0.18

Note: Using Student's *t*‐test and *Mann–Whitney *U* test.

Abbreviations: BMD, bone mineral density; CI, confidence interval; L, lumbar vertebrae; SD, standard deviation.

### 3.3 Factors related to low BMD in adults with CHD

**Figure 2:**
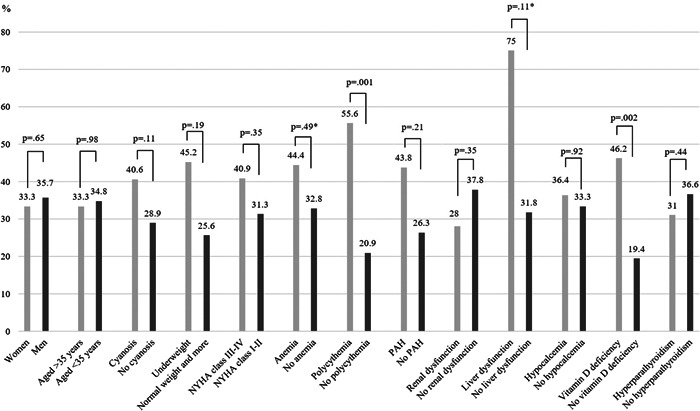
Prevalence of low bone mineral density for whole body by subgroups in adults with congenital heart disease, using *χ* ^2^ test or *Fisher's exact test. NYHA, New York Association; PAH, pulmonary artery hypertension.

Figure 2 shows the difference in the overall prevalence of low BMD between subgroups. Low BMD was more prevalent in patients with polycythemia than in those without (55.6% vs. 20.9%, *p* = 0.001) and in patients with vitamin D deficiency than in those without (46.2% vs. 19.4%, *p *= 0.002). The relationship between BMD and age in patients with CHD is described in Supporting Information: Table 2. We noted a moderated effect size of age to BMD at lumbar vertebrae 2 and lumbar vertebrae 3 in men (*R* = 0.505, *p* = 0.004 and *R *= 0.503, *p* = 0.004, respectively). Meanwhile, at other sites in both sexes, age‐related BMD was low. The results of the stratified logistic regression for predicting low BMD are summarized in Table 3. Multivariable logistic regression analysis showed that low BMD was associated with polycythemia (odds ratio, 4.72; 95% CI, 1.64–13.58; and *p *= 0.004).

**Table 3:** Stratified logistic regressions predicting low bone mineral density in adults with congenital heart disease

Independent variables	*β*	SE	*p* (Wald statistic)	OR (95% CI)
*Univariable logistic regression*				
Women	−0.105	0.513	0.84	0.9 (0.33; 2.46)
Age > 35 years	−0.065	0.532	0.9	0.94 (0.33; 2.66)
Cyanosis	0.518	0.507	0.31	1.68 (0.62; 4.54)
Underweight	0.871	0.515	0.09	2.39 (0.87; 6.55)
NYHA Class III–IV	0.421	0.534	0.43	1.52 (0.54; 4.34)
Anemia	0.495	0.724	0.5	1.64 (0.4; 6.78)
Polycythemia	1.552	0.539	0.004	4.72 (1.64; 13.58)
PAH	0.778	0.513	0.13	2.18 (0.8; 5.95)
Renal dysfunction	−0.445	0.541	0.41	0.64 (0.22; 1.85)
Liver dysfunction	1.861	1.185	0.12	6.43 (0.63; 65.53)
Hypocalcemia	0.134	0.539	0.8	1.14 (0.4; 3.29)
Vitamin D deficiency	1.273	0.557	0.02	3.57 (1.2; 10.63)
Hyperparathyroidism	−0.248	0.516	0.63	0.78 (0.28; 2.14)
*Multivariable logistic regression*				
Step 1				
Polycythemia	1.552	0.539	0.004	4.72 (1.64; 13.58)
Constant	−1.329	0.375	<0.001	

Abbreviations: CI, confidence interval; NYHA, New York Heart Association; OR, odds ratio; PAH, pulmonary artery hypertension; SE, standard error.

## 4 DISCUSSION

To the best of our knowledge, this study provided previously unreported evidence on the significant prevalence of low BMD in adults with CHD in Vietnam. Low BMD was more prevalent in patients with polycythemia than in those without, and vitamin D deficiency was prevalent in patients with the aforementioned health conditions. Moreover, polycythemia was an independent risk factor for low BMD in adults with CHD.

### 4.1 Patient characteristics

Patients were predominantly female with simple cardiac malformations, similar to a previous report.^10^ In developed countries, cardiac surgery and intervention have been routinely implemented to improve the outcomes of children with CHD, with an increasing number of patients surviving to adulthood. However, in many developing countries, repair and/or comprehensive treatment for CHD remains limited, explaining the common phenomenon of cardiac and noncardiac complications related to longstanding alterations in hemodynamics, neurodevelopment, and psychosocial development.^11^ Our patients presented with a high rate of complications, such as PAH, NYHA Class III‒IV, cyanosis, and polycythemia, suggesting an underdiagnosis and undertreatment of adults with CHD in Vietnam, which is similar to the situation in other developing countries.^12^ Notably, chronic hypoxia occurs in adults with cyanotic CHD, leading to an adaptive physiological polycythemia for increasing oxygen carrying capacity. Chronic hypoxia stimulates renal release of erythropoietin, resulting in increased production of red blood cells in the bone marrow and in the development of secondary erythrocytosis.^13^ This unique pathophysiology highlights the need for longstanding and multisystem management of CHD adults with polycythemia, such as care for bone‐related hematological complications. The high prevalence of vitamin D deficiency, hypocalcemia, and secondary hyperparathyroidism in adults with CHD was consistent with that reported by Izumi et al.^14^ Vitamin D synthesis in the skin upon natural sunlight exposure is the main source of vitamin D, while some foods and vegetables provide approximately 10% of vitamin D requirements. Thus, in the adult population of Vietnam, a tropical country with abundant sunshine throughout the year, vitamin D deficiency and secondary hyperparathyroidism should be less prevalent. Notably, adults with CHD had a low prevalence of severe vitamin D deficiency, but vitamin D deficiency and hyperparathyroidism were common in approximately one‐half and one‐third of patients, respectively. This finding regarding vitamin D is consistent with that of a recent observational study in the adult Vietnamese population^15^ and might be explained by the cultural norm of avoiding sunlight exposure, particularly among young Vietnamese people. Interestingly, the mean 25[OH]D and total calcium serum levels in our patients seemed be lower than those in the general adult Vietnamese population, although the mean serum PTH levels seemed higher.^15^ Therefore, adults with CHD may have other factors that affect vitamin D deficiency. One possible mechanism underlying this phenomenon is reduced outdoor activities and limited sunlight exposure, leading to biosynthetic disturbances of vitamin D in the skin. Moreover, we believe that vitamin D deficiency is related to undernutrition, intestinal malabsorption, and other metabolic factors. Notably, we observed a significant prevalence of an underweight status in our patients, which is consistent with previous data on the CHD population.^16^ In adults with CHD, being underweight could be due to nutritional deficiencies related to various causes, such as an unbalanced diet, anorexia, malabsorption, or energy requirements to control infection because of a weakened immune system. In addition, hypocalcemia was common among the patients of this study (29.6%). This is potentially related to long‐term undernutrition, vitamin D deficiency, and hyperparathyroidism.

### 4.2 Bone mineral density

We found reduced BMD levels in the lumbar spine as measured by DXA in adults with CHD, with no sex‐related differences in mean BMD at any site, except the trochanteric femur. It was previously reported that bone health is linked to low BMD and cardiovascular disease.^17^ A recent study also reported a tendency of reduced BMD among adults with complex CHD.^6^ The early progressive loss of BMD during childhood in patients with cyanotic CHD resulted in one‐third of children with cyanotic CHD having osteopenia‐range BMD, with 14% having *Z*‐scores <−2.0.^18^ The long‐term accumulation of risk factors may explain the increased rate of osteoporosis in adults with CHD.

Furthermore, our study demonstrated that, at the lumbar spine, the mean BMD in adults with CHD seemed to be lower than the Vietnamese population reference ranges (men, 0.867 vs. 0.93 g/cm^2^, and women, 0.853 vs. 0.87 g/m^2^). The same trend was also noted in the mean *T*‐score and *Z*‐score at the lumbar spine.^7^ Based on either proximal femur or lumbar spine BMD, we identified significant rates of low bone quality in adults with CHD. Although our study included young patients with a mean age of 32.4 years, osteoporosis prevalence was similar to that of the reference older Vietnamese population (≥50 years), that is, one‐third of patients had osteoporosis in both sexes.^19^ Notably, our linear regression model showed weak relationships between age and BMD at most sites in both sexes. Current observations indicate that osteoporosis prevalence increases with age. Bone density tends to stay stable in young people. However, with increasing age, most bone sites trend toward reduced BMD because of negative bone remodeling, such as increasing bone resorption.^20^ The equal prevalence of osteoporosis in our younger patients and the older reference population and weak effect size of age to BMD suggest that, in addition to age, there are hidden risk factors for osteoporosis in adults with CHD.

We found discordance in BMD at different sites, which suggested that some parts of the body had more bone loss and osteoporosis than others. The prevalence of low BMD in the lumbar spine was several times higher than that in the proximal femur, which is consistent with different populations.^21^, ^22^ For example, in a Canadian multicenter osteoporosis study, the prevalence of osteoporosis in women aged ≥50 years was 12.1% at the lumbar spine and 7.9% at the femoral neck.^22^ Undergoing DXA testing suggests that the increased bone loss in the lumbar spine compared with that in the proximal femur may be related to physiologic, pathophysiologic, and anatomic characteristics.^23^, ^24^ In adults, stable bone remodeling is covered by the delicate balance between bone resorption and formation. However, the periosteal apposition rate and net bone loss also depend on bone morphology, including cortical and trabecular compartments.^25^ The lumbar vertebrae needs to resist repetitive and high axial compression loads, whereas the proximal femur is mainly subjected to bending moments and shear forces. Correspondingly, bone location determines its characteristic microstructure. Cancellous bones at the core of the lumbar spine and proximal femur undergo remodeling and are metabolically active, making them sensitive to changes in bone resorption in osteoporosis.^26^ Moreover, the lumbar spine contains more cancellous bone than the proximal femur, suggesting that the lumbar spine undergoes more rapid deprivation than the proximal femur, leading to a higher risk of early osteoporosis.^26^

### 4.3 Factors related to low BMD

The prevalence of low BMD was significantly higher in adults with CHD than in those without vitamin D deficiency. Previous studies have reported a positive correlation between BMD and vitamin D, an important factor contributing to calcium absorption and bone metabolism.^27^ Prolonged vitamin D deficiency causes serious metabolic issues for bone health.^28^ Notably, low serum vitamin D levels is one of the signs for prolonged vitamin D deficiency. Moreover, serum vitamin D levels change during the year, with a significant decline in the percentage of adults with vitamin D deficiency being observed in the spring compared with that in the winter.^29^ This could explain the controversial results regarding the association between vitamin D deficiency based on low serum vitamin D levels and osteoporosis in recent studies. In our study, a significant relationship between vitamin D deficiency defined by low serum vitamin D levels and low BMD was found by univariable logistic regression, but not by multivariable logistic regression. In a study conducted in the Netherlands, vitamin D deficiency was associated with increased fracture risk in the population group aged 65–75 years but not in the older population group.^30^ Finally, a recent study in China also found low serum vitamin D levels in osteoporosis patients, but vitamin D deficiency was not an independent risk factor for low BMD.^31^

Secondary polycythemia was common in adults with CHD. However, osteoporotic conditions in patients with polycythemia have not yet been systematically studied. Using multivariable logistic regression, we confirmed that the likelihood of low BMD was greater in adults with CHD and coexisting polycythemia than in those without. Recently, evidence from an animal model indicated a negative association between polycythemia and bone mass through bone homeostasis and remodeling.^32^ In agreement with the microcomputed tomography, alterations in femoral bone remodeling were affected by polycythemia, including decreased bone volume ratio, trabecular numbers, osteoblast numbers, and osteoblast surface per bone surface. Additionally, the mice group with polycythemia had significantly lower osteoblast activity based on bone formation rate per tissue volume than the control mice group without polycythemia. Polycythemia in adults with CHD is the result of chronic hypoxic conditions that play a role in modulating bone cell responses by inhibiting osteoblast differentiation, reducing bone formation, and reducing osteoblast matrix mineralization.^33^ In a Danish population‐based cohort study, the risk of osteoporotic fractures among patients with polycythemia vera was significantly higher than in the general population.^34^ Patients with chronic hypoxic status have pathophysiological responses leading to increased production of red blood cells caused by elevated serum erythropoietin levels.^35^ Moreover, a recent study demonstrated that erythropoietin could stimulate osteoclast precursors toward increased bone resorption and reduced bone formation rate, thereby causing bone loss.^36^

We found trends of low BMD in patients with other risk factors for osteoporosis, such as cyanosis, underweight status, NYHA Class III‒IV, anemia, PAH, liver dysfunction, hypocalcemia, and hyperparathyroidism, although these factors were not statistically significant after a multivariable logistic regression analysis. Notably, hypoxia affected the function and formation of osteoclasts, the cells responsible for bone resorption, due to an increase extracellular anaerobic metabolism, causing osteoclast activation and stimulating osteoclast formation and resorption, leading to bone loss.^37^, ^38^ However, our study did not find an independent association between cyanosis and low BMD, similar to two recent studies in adults with complex CHD.^6^, ^39^ We suggest that a high number of patients with low SPO_2_ before intervention might have influenced the findings of these studies. Additionally, the long‐term effects on BMD depend on hypoxia. However, the median time until major intervention for patients with CHD in a study by Johansson et al.^39^ was 2.5 years, rendering them less hypoxic; this might explain the lack of association between current oxygen saturation and low BMD. Importantly, cyanosis and polycythemia are signs of the same pathophysiology status of CHD. Perhaps, in our study, the effect of polycythemia to osteoporosis had a masked effect of cyanosis to osteoporosis. Therefore, further studies are needed to elucidate the causative link between cyanosis and bone development and quality in adults with CHD.

### 4.4 Limitations

This study has some limitations. This was a cross‐sectional study, meaning that it cannot prove the causality between low bone quality measured by BMD and biological characteristics in adults with CHD. BMD reflects the cumulative effects of threats to bone health. Therefore, case‐control studies and longitudinal cohort studies are needed to identify disease‐specific risk factors as well as major consequences, including fractures and mortality. Second, this study was performed at a cardiovascular referral hospital in Vietnam, which might have caused sampling bias, and might have affected generalizability of the study findings. Additionally, we did not assess some candidate factors for low BMD, such as physical activity limitations and other biomarkers, which are common in adults with CHD. Regarding other known risk factors, no patients in our study reported a history of smoking, alcoholism, diabetes, or taking anticoagulants. We also missed collecting information on the use of other drugs, and diet and nutrition, which are important factors affecting BMD. Future investigations should focus on adults with CHD by combining all these factors. Finally, some subgroups were small, which might have affected our statistical analysis.

## 5 CONCLUSION

Our study revealed reduced BMD and a significant prevalence of low BMD in adults with CHD in Vietnam. These findings highlight the association between low BMD and biological characteristics, particularly polycythemia, in these patients. We recommend that adults with CHD, particularly those presenting the abovementioned risk factors, be screened, diagnosed, and monitored for adequate bone health.

## AUTHOR CONTRIBUTIONS

**Thanh‐Huong Truong**: Conceptualization; formal analysis; funding acquisition; writing – original draft; and writing – review and editing. **Mai‐Ngoc Thi Nguyen**: Funding acquisition; and writing – review and editing. **Ngoc‐Thanh Kim**: Formal analysis; writing – original draft; and writing – review and editing. **Thuy‐Hoa Thi Nguyen**: Formal analysis; and writing – review and editing. **Doan‐Loi Do**: Funding acquisition; and writing – review and editing. **Thanh‐Tung Le**: Writing – review and editing. **Hong‐An Le**: Writing – review and editing. All authors have read and approved the final version of the manuscript. Thanh‐Huong Truong had full access to all data in this study and takes complete responsibility for the integrity of the data and the accuracy of the data analysis.

## CONFLICT OF INTEREST

The authors declare no conflict of interest.

## TRANSPARENCY STATEMENT

Thanh‐Huong Truong affirms that this manuscript is an honest, accurate, and transparent account of the study being reported; that no important aspects of the study have been omitted; and that any discrepancies from the study as planned (and, if relevant, registered) have been explained.

## ETHICS STATEMENT

The protocol of the current study was approved by the Science Board and Ethics Committee of the Bach Mai Hospital (Number: 3758/QD‐BM; ID: BM_2020_1389). The study complied with the principles of the Declaration of Helsinki. All participants provided written informed consent.

## Supporting information

Supporting information.

Supporting information.

## Data Availability

The data that support the findings of this study are available from the corresponding author upon reasonable request.
