# Supplementary material for: Low bone mineral density and its related factors in adults with congenital heart disease in Vietnam: A cross‐sectional study
Source: Health Sci Rep. 2022 Aug 7;5(5):e732. doi: 10.1002/hsr2.732 (PMC9358147; doi:10.1002/hsr2.732)
Supplement: Supplementary file 1 — Supporting information. [file HSR2-5-e732-s002.docx]

**Supplementary Table 1 Types of congenital heart disease**

| **Anatomical defects, n (%)** | **% (n)** |
| --- | --- |
| ***Simple defects*** | ***71.2 (52)*** |
| Isolated ASD | 34.2 (25) |
| Isolated VSD | 19.2 (14) |
| Isolated PDA | 9.6 (7) |
| Isolated PS | 2.7 (2) |
| CAVc | 1.4 (1) |
| VSD + PDA | 2.7 (2) |
| Lutembacher syndrome | 1.4 (1) |
| ***Complex defects*** | ***28.8 (21)*** |
| Tetralogy of Fallot | 13.7 (10) |
| PA/IVS | 5.5 (4) |
| Single ventricle | 2.7 (2) |
| Ebstein’s anomaly | 1.4 (1) |
| DORV | 1.4 (1) |
| cTGA+ ASD | 1.4 (1) |
| Tricuspid atresia | 1.4 (1) |
| ALCAPA | 1.4 (1) |
| ALCAPA: Anomalous left coronary artery from the pulmonary artery; ASD: Atrial septal defect; CAVc: Complete atrioventricular septal defect; DORV: Double outlet right ventricle; PA/IVS: Pulmonary atresia with intact ventricular septum; PDA: Patent ductus arteriosus; PS: Pulmonary stenosis; cTGA: Corrected transposition of great arteries; VSD: Ventricular septal defect. | |
