# Supplementary material for: Low bone mineral density and its related factors in adults with congenital heart disease in Vietnam: A cross‐sectional study
Source: Health Sci Rep. 2022 Aug 7;5(5):e732. doi: 10.1002/hsr2.732 (PMC9358147; doi:10.1002/hsr2.732)
Supplement: Supplementary file 2 — Supporting information. [file HSR2-5-e732-s001.docx]

**Supplementary Table 2 Linear regression was used to model the relationship between age and BMD at the lumbar spine and left proximal femur for adults with congenital heart disease**

| **Site** | **Total** | | **Women** | | **Men** | |
| --- | --- | --- | --- | --- | --- | --- |
|  | ***R*** | ***p*** | ***R*** | ***p*** | ***R*** | ***p*** |
| ***Lumbar spine*** | | | | | | |
| *Lumbar vertebrae 1* | 0.042 | .72 | 0.197 | .21 | 0.324 | .08 |
| *Lumbar vertebrae 2* | 0.104 | .38 | 0.229 | .14 | 0.505 | .004 |
| *Lumbar vertebrae 3* | 0.088 | .46 | 0.25 | .11 | 0.503 | .004 |
| *Lumbar vertebrae 4* | 0.073 | .54 | 0.324 | .04 | 0.21 | .26 |
| *Full* | 0.027 | .82 | 0.271 | .08 | 0.406 | .02 |
| ***Left proximal femur*** | | | | | | |
| *Neck* | 0.101 | .39 | 0.067 | .67 | 0.158 | .4 |
| *Troch* | 0.027 | .82 | 0.178 | .26 | 0.203 | .27 |
| *Inter* | 0.026 | .83 | 0.284 | .07 | 0.183 | .33 |
| *Full* | 0.058 | .63 | 0.222 | .16 | 0.296 | .11 |
